# Habitat selection by a threatened desert amphibian

**DOI:** 10.1002/ece3.7074

**Published:** 2020-12-09

**Authors:** Ross K. Hinderer, Andrea R. Litt, Magnus McCaffery

**Affiliations:** ^1^ Department of Ecology Montana State University Bozeman MT USA; ^2^ Turner Endangered Species Fund Bozeman MT USA

**Keywords:** arid landscapes, conditional logistic regression, desiccation risk, fragmentation, *Lithobates chiricahuensis*, microclimate, resource selection

## Abstract

Habitat degradation and fragmentation are major drivers of amphibian declines. The loss of environmental features that allow for movement between water sources may be particularly detrimental for amphibians in arid environments. Climate changes will increase the importance of microhabitats to amphibians. Enhancing areas to facilitate movement may be a necessary conservation strategy for many animal species that depend on wetlands, including federally threatened Chiricahua leopard frogs (*Lithobates chiricahuensis*). Habitat preferences of this frog species are not well understood. We sought to better understand fine‐scale habitat selection, to inform conservation of Chiricahua leopard frogs.

We conducted our study on the Ladder Ranch, a privately owned working bison ranch in New Mexico, USA that supports a large proportion of the remaining Chiricahua leopard frogs in the state.

We attached radio transmitters to 44 frogs during summer 2014. We located each frog daily for up to 8 weeks (median = 30 days). We assessed fine‐scale habitat selection by comparing characteristics at each frog location and a random location 5 m away using conditional logistic regression.

Frogs preferred features that likely reduce desiccation, even after accounting for the presence of water. Frogs selected areas with more low‐lying cover, especially aquatic vegetation and woody debris, a tree overstory, and a mud substrate.

We recommend managing potential movement corridors for Chiricahua leopard frogs by ensuring the presence of muddy creek bottoms, woody debris, riparian overstory, low‐lying ground cover, and pools. Microclimates created by these features seem especially valuable given warming temperatures and modified precipitation regimes, resulting in decreased surface water, soil moisture, and vegetation cover. Retaining or creating preferred habitat features and microclimates in areas between water sources may increase connectivity among isolated populations of Chiricahua leopard frogs and could improve persistence and recovery of other water‐obligate species in arid landscapes.

## Graphical abstract

Retaining or creating preferred habitat features and microclimates in areas between water sources may increase connectivity among isolated populations of Chiricahua leopard frogs and could improve persistence and recovery of other water‐obligate species in arid landscapes. We studied the habitat preferences of Chiricahua leopard frogs as they moved between water sources and present those selection results in order to better inform management of habitat for this threatened amphibian.

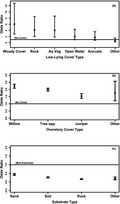

## 1 INTRODUCTION

In the last worldwide assessment of amphibian species status, Stuart et al. (2004) estimated that more than 32% of amphibians were threatened with extinction, though other assessments suggest that number may be as high as 53% of known species (International Union for the Conservation of Nature & Natural Resources, 2018). Recent work predicts that half of the amphibian species lacking sufficient data on current status may also be threatened with extinction (González‐del‐Pliego et al., 2019). Many mechanisms have been proposed to explain declines, including climate change (Kissel et al., 2019; Wake & Vredenburg, 2008), solar UV‐B radiation (Blaustein et al., 1994), introduced predators (Adams, 2000; Pilliod & Peterson, 2001), infection by a pathogenic fungus (*Batrachochytrium dendrobatidis* [“*Bd*”], Scheele et al., 2019), and parasites (Leary et al., 2018). However, habitat destruction, degradation, and fragmentation are major causative agents of the observed trends (Green, 2005).

Because amphibians require moisture on their skin for effective respiration (Wake & Vredenburg, 2008), successful terrestrial movement is contingent on the availability and continuity of water sources or moist environments. The fate of amphibians under climate change scenarios will be based largely on characteristics of the habitat they occupy, and the spatial configuration of these habitat patches on the landscape (Opdam & Wascher, 2004).

The Southwestern United States has experienced drought conditions for much of the early 21st century; similar conditions are likely to persist into the future (Seager et al., 2007). In addition to the overall trend, droughts are likely to be longer and more severe (MacDonald et al., 2008) and decadal variations in precipitation are becoming more extreme (Sheppard et al., 2002), resulting in decreases in available water. Water sources also are expected to decline because of increased groundwater pumping to support a growing human population (Aeschbach‐Hertig & Gleeson, 2012). Wetlands in the Chihuahuan Desert ecoregion of the Southwestern United States and Mexico have been designated as one of the “Global 200” ecosystems of highest conservation concern (Olson & Dinerstein, 1998). More than 20% of desert wetlands in the Southwestern United States and northern Mexico no longer provide functioning habitat for wildlife (Minckley et al., 2013). Decreases in precipitation and increases in temperature contribute to a more fragmented landscape for amphibian species in semi‐arid ecosystems. Minckley et al. (2013) found that 19% of animal and plant species in Arizona listed under the Endangered Species Act (ESA) (or candidates for such listing) were associated directly with permanent desert wetlands; this list includes three amphibians. Because amphibians in the arid southwest may be particularly susceptible to habitat‐related changes, future management actions that actively mitigate effects of habitat loss or fragmentation are essential for amphibian conservation.

When the quantity or quality of natural water bodies declines, human‐subsidized aquatic resources, such as stock water tanks, can provide essential habitat for amphibians (Rosenstock et al., 1999). Conservation strategies for amphibians might require anthropogenic manipulation of water levels to mimic seasonal patterns that occurred prior to climatic changes (Shoo et al., 2011). However, conserving water in ponds (i.e., breeding habitat for many species) alone may not be sufficient to support foraging, movement, and hibernation of amphibians (Marsh & Trenham, 2001). In fact, habitat used during nonbreeding periods may be as important as breeding sites (Fellers & Kleeman, 2007). Studying where or how far an individual amphibian moves and the associated environmental features, such as type and amount of vegetation cover, may help us to better understand where individuals experience lower risk of desiccation (Cline & Hunter, 2014; Fellers & Kleeman, 2007; Mazerolle & Desrochers, 2005). With this information, active management practices could focus on providing important environmental features to enhance movement through areas between breeding ponds or other aquatic features.

The Chiricahua leopard frog (*Lithobates chiricahuensis*) is native to central and southeastern Arizona and Southwestern New Mexico, USA, and northern Mexico (Platz & Mecham, 1979; Stebbins, 1985). This species is highly aquatic and rarely found far from water (Clarkson & Rorabaugh, 1989; Sredl & Jennings, 2005). Chiricahua leopard frogs are found in natural streams with rocky pools, springs, and ponds, but man‐made livestock water tanks (which can include metal and earthen‐pond types) also provide important habitat (Stebbins, 1985). Anthropogenic water sources will potentially increase in importance to these frogs as groundwater pumping in the Southwestern United States dewaters natural desert springs (Unmack & Minckley, 2008). In 2002, Chiricahua leopard frogs were listed as threatened under the Endangered Species Act due in part to human‐caused habitat loss (USFWS, 2002). Although Chiricahua leopard frogs can generally colonize a range of aquatic features, competition and exclusion by introduced species has forced this frog into a much narrower realized niche of mostly ephemeral or inconsistent water sources with fewer aquatic cohabitants that rely on perennial water (USFWS, 2007). This species is affected by other threats common among amphibians, including *Bd* infection (Boykin & McDaniel, 2008) and introduced predators such as American bullfrogs (*Lithobates catesbeianus*, Rosen & Schwalbe, 1995). Chiricahua leopard frogs now are found in less than 25% of their historic range in the United States (USFWS, 2002) and have likely declined in Mexico (Rorabaugh et al., 2018).

Recovery of Chiricahua leopard frogs will be more likely when habitat patches are less fragmented and individuals can disperse long distances to reach breeding sites (Fellers & Kleeman, 2007). However, there is little published information about important habitat characteristics, especially outside of ponds. Understanding habitat use is crucial to conservation due to uncertainty about the future of water resources, the species’ limited range in the desert southwest, listing under the ESA, and the paucity of information available about the species’ habitat requirements.

To learn more about fine‐scale habitat selection between water sources, we set out to identify environmental characteristics Chiricahua leopard frogs select during the monsoon season, when landscapes may be more permeable to frog movement. Understanding fine‐scale selection processes will allow managers to maintain environmental features that facilitate frog movement within riparian corridors that are seasonally connected during monsoon events. Understanding habitat selection during movement is especially important in a human‐dominated landscape where connectivity of populations likely influences persistence (Ficetola & Bernardi, 2004). We hypothesized that selection of a specific location by a frog would be driven by factors that reduce the chances of desiccation, given that sources of standing water are relatively scarce in arid landscapes. We studied habitat selection by Chiricahua leopard frogs using radio telemetry and collecting information on the environmental characteristics of selected locations, relative to what was available in the area. We carefully selected explanatory variables for measurement and modeling based on literature on Chiricahua leopard frogs and related species (e.g., Blomquist & Hunter, 2009; Cline & Hunter, 2014; Howell et al., 2018; Jarchow et al., 2016; Mazerolle & Desrochers, 2005; Stebbins, 1985; Wallace et al., 2010) to characterize potential dessication and predation risk. Specifically, we considered the proximity to a water source, substrate type, and the type and amount of both low‐lying and overstory cover. We predicted that frogs would select locations with a nearby source of water, wet muddy substrates, and higher amounts of both low‐lying and overstory cover (Blomquist & Hunter, 2009; Howell et al., 2018; Mazerolle & Desrochers, 2005; Wallace et al., 2010).

## 2 METHODS

### 2.1 Study site

**FIGURE 1:**
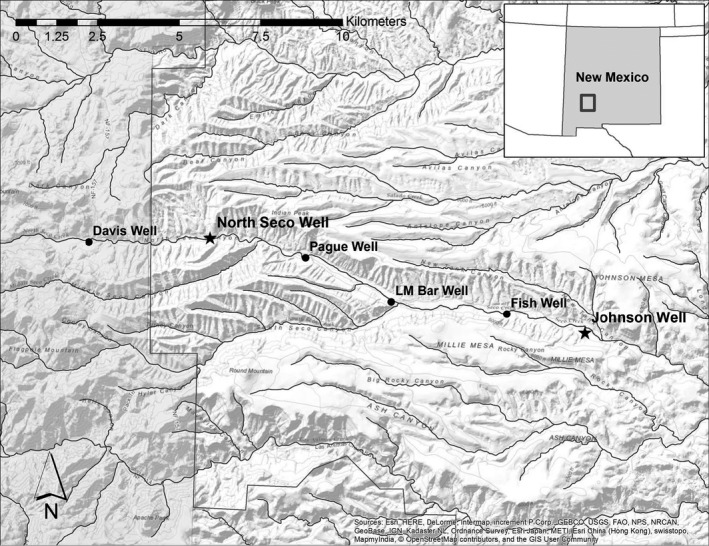
Layout of earthen‐pond livestock tanks on the Ladder Ranch, Sierra County, New Mexico, USA. Seco Creek is the seasonal drainage shown on the aerial imagery. Stars indicate focal sites for frog capture

We focused our research in Southwestern New Mexico, in the Arizona‐New Mexico Mountains ecoregion of the US and Mexico. Our study site was within a privately owned ranch (Ladder Ranch, 33°N, −107°W), which provides habitat for 33% of the known populations of Chiricahua leopard frogs in New Mexico (Kruse & Christman, 2005), making it a location of great conservation importance. Frogs utilize the complex of earthen‐pond and metal livestock watering tanks that are maintained via solar‐powered groundwater pumping on the ranch for American bison (*Bison bison*). Our focal sites for trapping frogs were two earthen‐pond tanks in the Seco Creek drainage (Figure 1). We conducted our research during the late summer monsoon season, as this is when Chiricahua leopard frogs are thought to disperse among the perennial water sources where they breed (USFWS, 2007). Outside of monsoon season, there is very little to no standing water within the ephemeral stream channel of the Seco Creek drainage where our focal earthen‐pond tanks were located, although during monsoons water may flow and pools may remain in the stream channel between storms (R. Hinderer, personal observation). The stream channel and nearby riparian habitat create the assumed travel corridor for frogs moving between perennial water sources within Seco Creek drainage (Kruse & Christman, 2005).

### 2.2 Frog capture

To capture frogs, we encircled two earthen‐pond tanks with drift fences and pitfall traps (Dodd & Scott, 1994). From 22 June to 5 August 2014, we checked pitfall traps twice daily as weather permitted. In addition, we captured frogs opportunistically in the Seco Creek stream channel using dip nets.

### 2.3 Radio telemetry

We selected animals for radio telemetry where the transmitter did not exceed 10% of the animal's mass (Richards et al., 1994) and attempted to select animals from both sexes and all size classes that met the mass criteria. Blomquist and Hunter (2007) found transmitters up to 10% of frog body mass had little effect on the vagility of northern leopard frogs (*Lithobates pipiens*), a closely related species of a similar size (Stebbins, 1985). We attached a transmitter (Holohil Systems model BD‐2, 0.62, 0.9, or 1.2 g, www.holohil.com) to frogs following the method of Muths (2003), stringing a fine piece of elastic cord through glass beads to build a belt placed around the frog's waist. We located animals every day or as monsoon storm conditions allowed from 4 July to 7 August 2014, tracking frogs until we established a visual ID of the animal or to within a small radius if not visible (~0.1 m).

### 2.4 Habitat data

Each time, we located a transmittered frog, we recorded the time, Universal Transverse Mercator (UTM) location, and several ecological characteristics (Table 1). As Chiricahua leopard frogs are vulnerable to desiccation (Howell et al., 2018) and predation by a variety of birds, mammals, and reptiles (Sredl & Jennings, 2005), we collected data on covariates that characterized desiccation and predation risk: presence of a water source, the substrate, and the vegetative cover at ground level (low‐lying) and above (overstory) the frog's location. We recorded the presence of water at the frog's location (pooled or flowing water; present/absent). We visually estimated the amount and type of “low‐lying cover” (any vegetation or other cover, e.g., woody debris, undercut stream banks), the amount and type of overstory cover, and the dominant substrate type within a 1‐m diameter circle centered on the frog's location. We quantified the same characteristics at a random location, 5 m away from the frog at a random compass bearing (1–360°). We assumed this location was accessible and available to a frog, but far enough away to be selected differently. Collecting data in this way resulted in pairs of locations, one where the frog was found (the “selected” location) and another that was “available” to the frog, but not occupied (Compton et al., 2002). The scale of resource selection functions is essential for relevant inferences (Boyce, 2006) and fine‐, rather than broad‐scale characteristics may be important in habitat selection by amphibians (Gorman & Haas, 2011). Comparing the paired selected and available locations allowed us to quantify characteristics important to frogs on a fine scale.

**TABLE 1:** Variables measured at Chiricahua leopard frog locations and random locations, Sierra County, New Mexico, USA, summer 2014

Variable	Definition^a^
WATER	Presence of water at the frog or random location, yes or no
DISTANCE	Distance (in m) to the nearest source of water (not used in analysis)
SUBSTRATE	Most common substrate type—mud, sand, rock, soil, or other
LCOVERPCT	Percentage (0%–100%) of low‐lying cover a frog could use to hide from predators
LCOVERTYP	Most common type of low‐lying cover—none, annuals, open water, rock, aquatic vegetation, woody cover, or other
OCOVERPCT	Percentage (0%–100%) of overstory cover above (not used in analysis, converted to OCOVERPRES, below)
OCOVERTYP	Most common type of overstory cover—none, juniper, willow, tree spp., or other
OCOVERPRES	A categorical indicator for whether there was >10% overstory cover, yes or no

^a^ Excluding WATER and DISTANCE, all variables were collected within a 1 m diameter circle centered on the location.

### 2.5 Analysis

Prior to analysis, we examined the distribution of possible explanatory variables. We condensed substrate and cover types that we documented infrequently to an “other” category. We maintained separate categories of overstory type for juniper and willow, which were used commonly, but condensed all other tree species into a general “tree spp.” category. More than half of all locations were in areas without overstory cover (65%, 1,348 out of 2,072 total observations), limiting our ability to make inference across the range of this covariate. As such, we converted the percentage of overstory cover at the location into a categorical variable with two levels, little to no overstory cover (≤10%) or greater overstory cover (>10%), which we included as a potential covariate.

We analyzed the importance of these covariates to frog habitat selection with a conditional logistic regression model using Cox proportional hazards (Compton et al., 2002; Poole et al., 2009; Popescu et al., 2013; Zeller et al., 2014) in the *coxme* package in R (R Core Team, 2014). This model formulation allows the explicit pairing of observations to match our data collection, where selection is conditional on what is available to each individual at a specific time. The approach is analogous to the case‐control study frequently used in epidemiology (see Breslow, 1996; Buerhing et al., 2015; Ström et al., 2017). To account for variation in the number of observations and potential differences in habitat selection among frogs, we included a random intercept for each individual frog (Duchesne et al., 2010).

We selected an inferential model based on the stepwise variable selection method of Hosmer and Lemeshow (2000). This method begins with the assumption that all explanatory variables available may be important and then tests the explanatory value of those variables sequentially during the formulation of an inferential model. Advantages to this sequential process include the testing of individual terms during model building rather than ranking models that represent alternative hypotheses, the formulation of which would be difficult for such a little‐studied species (see Compton et al., 2002). We carefully considered covariates to explore dessication risk based on previous studies and assumed all of these could be important. The initial model included all single terms (Table 1). We reached a tentative additive model by eliminating single variables that did not help explain variation in selection and tested interactions between remaining terms. We tested all interactions one at a time by adding them to the model, with one exception. When low‐lying cover was absent, we could not assign a cover type, such that the interaction between cover type and amount of cover was inestimable. We also examined evidence for a quadratic relationship between selection and percentage of low‐lying cover, as we hypothesized that frogs may prefer an intermediate level of cover. We used likelihood‐ratio tests to compare nested models, examining chi‐square statistics to compare models that differed by a discrete variable and *t* tests to compare models that differed by a continuous variable. We removed terms that did not explain sufficient variation (*p* > 0.1 from a likelihood‐ratio test or *t* test) or where a model failed to converge.

## 3 RESULTS

We tracked 44 Chiricahua leopard frogs (15 male, 13 female, 16 immature; snout‐urostyle length range = 44–95 mm) using radio telemetry, locating frogs a total of 1,036 times. The duration of the tracking period varied among individuals (range = 1–66 days, median = 30 days). Frogs preferentially selected locations based on presence of water, percent cover (quadratic relationship), and type of cover available to a frog, type of overstory cover, and substrate (Table 2) but did not select locations based on the amount of overstory cover (categorized as ≤ or >10% cover). We present results as odds ratios, which express the difference in odds between the selection of a site with the variable present versus a baseline (for categorical variables) or between the selection of a site where the variable level increases versus a baseline of zero (for the continuous variable).

**TABLE 2:** Likelihood‐ratio tests comparing the inferential model (where we accounted for WATER, LCOVERPCT, LCOVERPCT^2^, LCOVERTYPE, OCOVERTYPE, and SUBSTRATE) to the same model without the specified term

Variable	*df*	*χ* ^2^	*p*
WATER	1	24.76	<0.001
LCOVERPCT	1	67.78	<0.001
LCOVERPCT^2^	1	48.42	<0.001
LCOVERTYPE	6	61.11	<0.001
OCOVERTYPE	4	17.78	0.001
SUBSTRATE	4	43.58	<0.001

### 3.1 Presence of water

Frogs were 2.92 (95% CI = 2.78–3.07) times more likely to select a location with water, compared to one without.

### 3.2 Low‐lying cover

**FIGURE 2:**
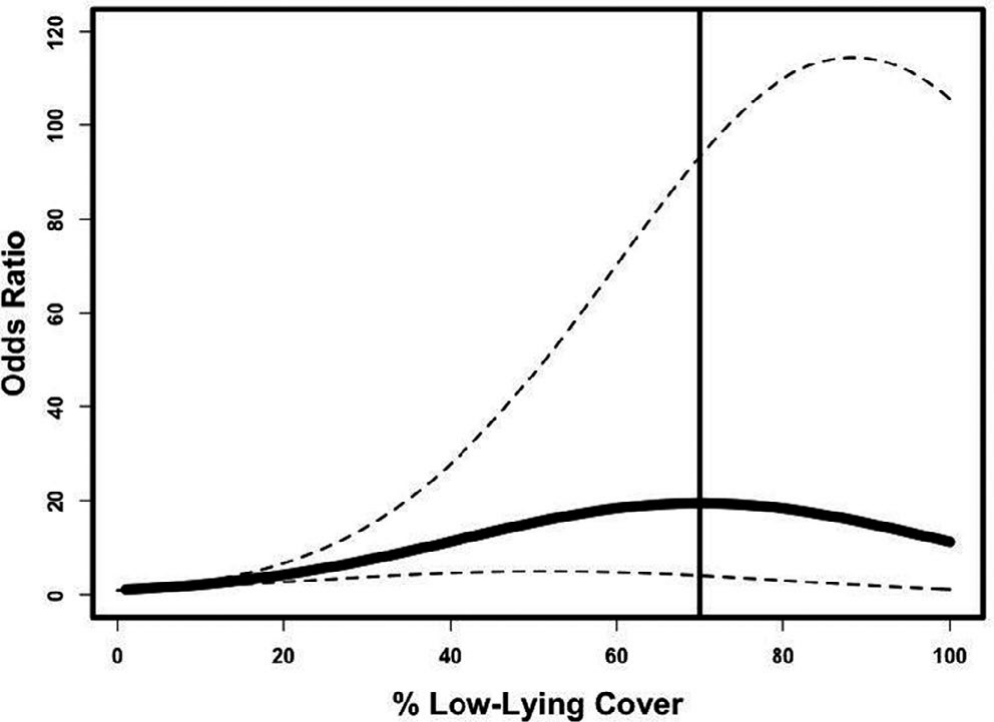
Odds ratios of a frog selecting a location with different amounts of low‐lying cover, compared with a location with 0% cover (where odds ratio = 1). Selection was maximized at 70% cover (indicated by vertical line). Dashed lines indicate 95% confidence interval

**FIGURE 3:**
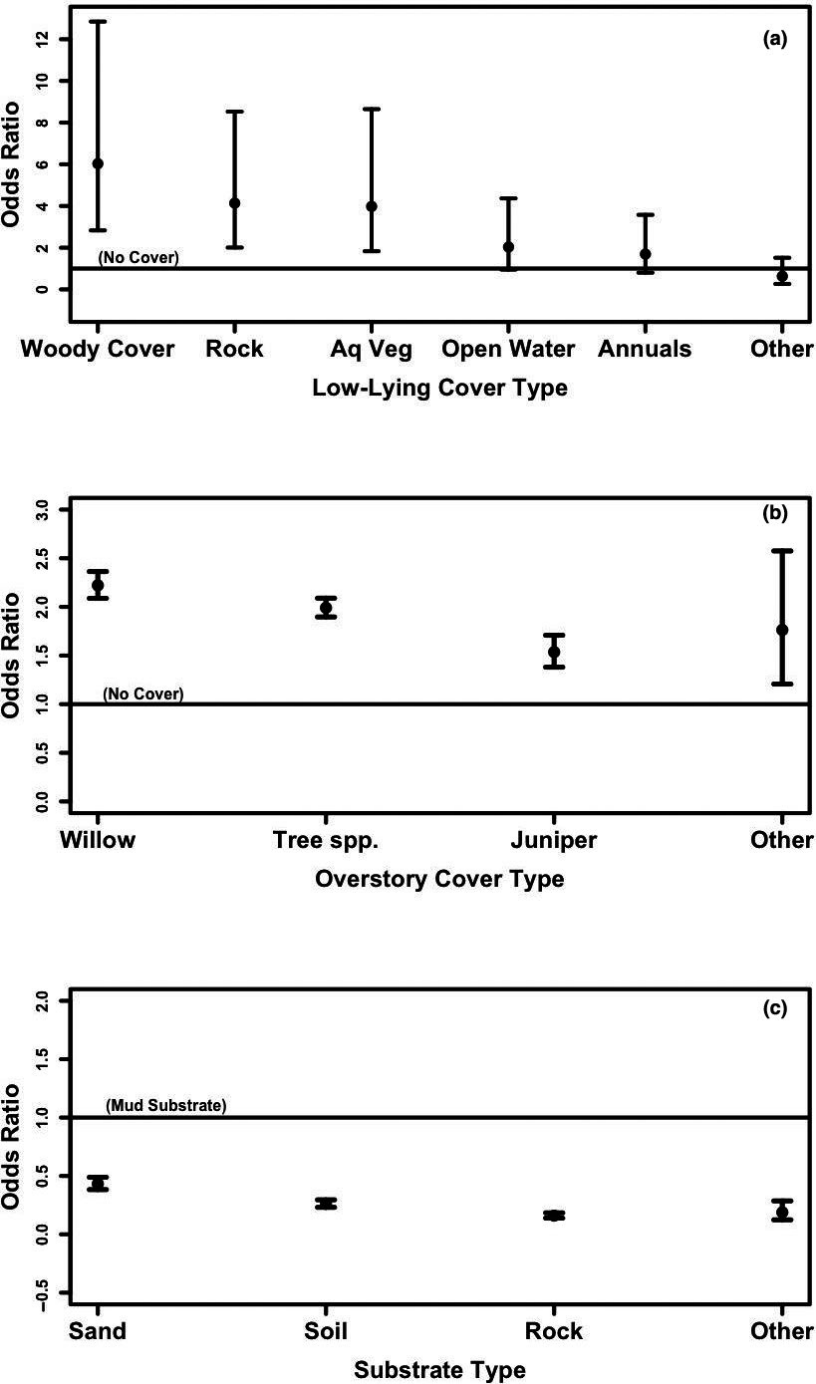
Odds ratios (and 95% confidence intervals) of a frog selecting a location with different (a) types of low‐lying cover, (b) types of overstory cover, and (c) types of substrate, compared with a location without low‐lying or overstory cover, and a mud substrate (reference line where odds ratio = 1). Odds ratios < 1 indicate that frogs were less likely to select substrates other than mud. Note difference in the scale of the *y*‐axes

Compared with a location with no cover, frogs were 19.49 (4.08–93.22) times more likely to select a site within a 1 m circle that provided more low‐lying cover. Selection probability increased to a maximum of 70% cover, although uncertainty around this estimate was high (Figure 2). Frogs were more likely to select locations with any type of cover compared with no cover at all (Figure 3a). Specifically, frogs were 6.03 (2.83–12.85) times more likely to select woody cover, 4.14 (2.01–8.53) times more likely to select rock, and 3.98 (1.83–8.65) times more likely to select aquatic vegetation compared to a location with no cover. We also found some evidence that open water (2.03 times, 0.95–4.37) and annual plants (1.69 times, 0.80–3.58) were preferred to no cover (Figure 3a).

### 3.3 Overstory cover

Compared with a location with no overstory, frogs were more likely to select a location within a 1 m diameter circle with any type of overstory cover (Figure 3b). Frogs were 2.22 (2.09–2.36) times more likely to select a location with a willow overstory, 1.99 (1.90–2.09) times more likely to select a location with an overstory of trees other than juniper or willow, and 1.54 (1.38–1.71) times more likely to select a juniper overstory, compared with no overstory at all.

### 3.4 Substrate

Frogs were more likely to select mud compared to any other substrate (Figure 3c). Frogs were 0.43 (0.38–0.49) times as likely to select sand, 0.26 (0.23–0.30) times as likely to select soil, and 0.16 (0.14–0.18) times as likely to select rock, compared with a mud substrate.

## 4 DISCUSSION

Our study identified habitat characteristics important for Chiricahua leopard frogs during the summer monsoon season, adding to the limited knowledge about a period when frogs are more likely to move out of perennial ponds (USFWS, 2007). We found that characteristics that lessen the risk of desiccation (low‐lying cover, overstory cover, and mud substrate) were important for selection by Chiricahua leopard frogs, even after accounting for presence of water. Similarly, Blomquist and Hunter (2009) found that northern leopard frogs selected locations based on proximity to standing water and greater amounts of low‐lying cover, in a managed forest in Maine. Lowland leopard frogs (*Lithobates yavapaiensis*), a closely related species found in similar arid environments, selected pools with more vegetation, more overstory cover, and greater heterogeneity around the edges of pools (Wallace et al., 2010). Given that adult Chiricahua leopard frogs are highly aquatic, water is clearly important for this species. However, due to the scarcity of standing water in arid landscapes, desert amphibians must often rely on other environmental features to decrease the risk of desiccation.

**FIGURE 4:**
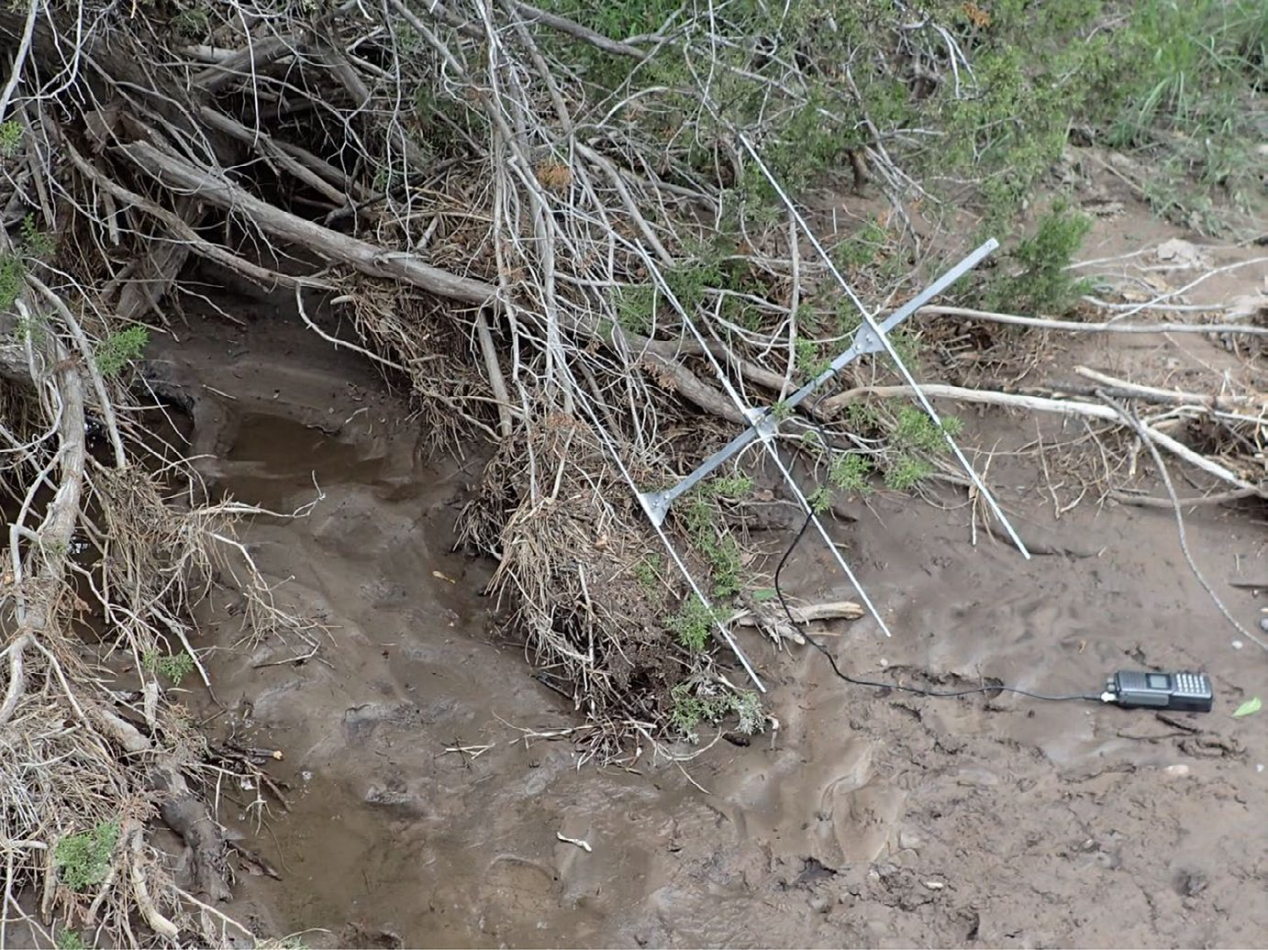
An example of typical monsoon‐created Chiricahua leopard frog habitat. Note the woody cover, juniper overstory, and mud substrate. The receiver antenna points to the location where we found a frog

Our study is the first to focus on environmental features selected by Chiricahua leopard frogs at a fine scale; we think this approach can identify potential foci for conservation or restoration efforts. Based on our results, we recommend that managers enhance or maintain a moderately high percentage of low‐lying cover comprised of woody debris, aquatic vegetation, and rock, a tree overstory, and a mud substrate to provide habitat for Chiricahua leopard frogs outside of ponds where they breed. Perhaps unsurprisingly, these are similar to features created by floods resulting from summer monsoons (Figure 4). High flows create piles of woody debris providing cover and deposit water‐retaining mud in stream bottoms that otherwise may be dry for most of the year. We propose that even in the absence of water, features created by monsoon flows reduce the potential for desiccation and may provide important habitat for Chiricahua leopard frogs. Similarly, the understory composition of Florida pine plantations mediates the response of amphibians to drying, with Southern toads (*Anaxyrus terrestris*) experiencing greater desiccation risk in areas with less herbaceous groundcover and moving through more densely covered areas when available (Haggerty et al., 2019). Low‐lying cover could also provide some protection from predation. To improve habitat features important for Chiricahua leopard frogs, we suggest that managers pay special attention to micro‐environments created by monsoon flows and avoid activities that reduce these features, such as cutting riparian overstory cover, removing deadfalls and woody debris from streams, dredging creek or canal channels, or mowing potential movement corridors.

Movement between sources of water allows genetic exchange that is important for population persistence (Bowler & Benton, 2005), yet the composition of the matrix between water sources can change movement rates of amphibians (Eycott et al., 2012). Variation in permeability of different matrix types differs from the classic dichotomous classification of core amphibian habitats (e.g., ponds) embedded within a matrix of nonhabitat that may not reveal features of fine‐scale heterogeneity important to amphibians (Pope et al., 2000). Our study identifies important characteristics of this fine‐scale heterogeneity, which can be useful in further studies that seek to develop a patch‐matrix view of amphibian movement. Howell et al. (2018) examined movement of Chiricahua leopard frogs in Arizona, finding that slope and distance to the nearest streambed were important characteristics in predicting colonization rates of stock tanks. We suggest that using individually modeled selection for specific habitat characteristics can improve the assumptions of movement studies and increase the overall utility of those data for conservation by allowing more specific recommendations about preferred landscape features.

Chiricahua leopard frogs currently occupy an inherently fragmented landscape of livestock watering tanks and perennial pools (Stebbins, 1985) that are tenuously connected by ephemeral and unpredictable stream channels. This is an increasingly common scenario for amphibians in arid environments in the face of changes in land use and climate. Maintaining habitat connectivity is an essential component of an effective amphibian management plan (Semlitsch, 2000), and our findings about characteristics selected by Chiricahua leopard frogs could improve habitat for frogs moving between year‐round occupied ponds. Across North America, lower availability of water during breeding seasons will likely have an overwhelmingly negative effect on amphibian communities (Miller et al., 2018).

Human intervention may be needed to maintain habitat for desert amphibians in the face of a more arid climate (Seager et al., 2007). Groundwater pumping for use in agriculture and ranching will further deplete natural water resources (Aeschbach‐Hertig & Gleeson, 2012), making aquatic subsidies increasingly important for desert amphibians. Altering the amount of available water or the distances between sources of surface water may not always be possible due to limits imposed by regulations or human use. Under such conditions, enhancing characteristics of habitat between existing water sources could promote movement. The recovery plan for the Chiricahua leopard frog requires functioning metapopulations within recovery units to remove the threatened status but also cites a lack of understanding about movement abilities of this species (USFWS, 2007). Metapopulation function relies on the ability of animals to move between patches and to recolonize extinct patches (Levins, 1969). Although we did not quantify dispersal, our study identifies environmental features selected by Chiricahua leopard frogs during the time of year when dispersal is likely (USFWS, 2007). Long‐distance dispersing amphibians are those most likely to colonize extinct patches (Fellers & Kleeman, 2007) and disturbance or destruction of movement corridors may limit dispersal, with concomitant effects on population dynamics. Limiting anthropogenic disturbance around amphibian breeding and overwintering locations is appealing (e.g., Semlitsch & Bodie, 2003), but balancing the needs of amphibians with human activities is challenging in working landscapes where access to water is a limiting factor. For example, in the Southwestern United States, many water‐obligate species rely on tanks provided for livestock because surface water may be present for only part of the year (Rosenstock et al., 1999). By enhancing connectivity between water sources, managers may be able to augment wildlife habitat without totally curtailing appropriation of surface water for agriculture or other uses. Polasky et al. (2005) developed a model of habitat conservation that balances the needs of wildlife and economic productivity of working landscapes, but they point out the need for specific information on the requirements and movement abilities of the wildlife species of interest. Studies such as ours and a concurrent analysis of movement patterns (Hinderer et al., 2017) provide information useful for assessing the tradeoffs between economic and conservation goals in working landscapes.

Anthropogenic activities have altered the structure and function of nearly all ecosystems, to the point of modifying geologic processes (Steffen et al., 2007). Conservation in this new age will require human effort to mitigate or reverse environmental changes to preserve at‐risk species. More studies such as ours, in other systems and focusing on different species, will refine our understanding of the specific habitat requirements of animals affected by global climate change. Especially under climate change scenarios, human intervention will likely be required to “engineer a future” for amphibians in anthropogenic landscapes (Shoo et al., 2011). Understanding reactions to environmental conditions at a small scale is essential to understanding species’ responses to climate change effects (Hannah et al., 2014). In the case of the Chiricahua leopard frog, efforts to preserve or enhance habitat should focus on retaining low‐lying and overstory cover and muddy stream bottoms, even outside ponds. Preserving or restoring small pockets of habitat that mimic natural features could buffer populations against the effects of climate change by reducing desiccation and increasing permeability of landscapes to dispersing animals (Opdam & Wascher, 2004).

## CONFLICT OF INTEREST

The authors declare no conflict of interest.

## AUTHOR CONTRIBUTIONS

**Ross K. Hinderer:** Conceptualization (equal); data curation (lead); formal analysis (lead); investigation (lead); methodology (equal); writing – original draft (lead); writing – review and editing (equal). **Andrea R. Litt:** Conceptualization (equal); funding acquisition (equal); investigation (equal); methodology (equal); project administration (equal); resources (equal); supervision (equal); writing – original draft (supporting); writing – review and editing (equal). **Magnus McCaffery:** Conceptualization (equal); funding acquisition (equal); investigation (equal); methodology (equal); project administration (equal); resources (equal); supervision (equal); writing – original draft (supporting); writing – review and editing (supporting).

## Data Availability

Data used in this manuscript are deposited in Dryad at https://doi.org/10.5061/dryad.h9w0vt4gp.
